# Sleep Habits, Quality of Life and Psychosocial Aspects in the Older Age: Before and During COVID-19

**DOI:** 10.3389/fnins.2022.694894

**Published:** 2022-03-24

**Authors:** Katie Moraes de Almondes, Eleni de Araujo Sales Castro, Teresa Paiva

**Affiliations:** ^1^Neuropsychology of Ageing Service, Department of Psychology and Postgraduate Program in Psychobiology, AMBSONO Sleep Clinic, Onofre Lopes University Hospital, Federal University of Rio Grande do Norte, Natal, Brazil; ^2^Postgraduate Program in Psychobiology, Federal University of Rio Grande do Norte, Natal, Brazil; ^3^CENC –Sleep Medicine Center, Lisbon, Portugal; ^4^ISAMB – Faculdade de Medicina, Universidade de Lisboa, Lisbon, Portugal; ^5^CHRC – Comprehensive Health Research Center, Universidade Nova de Lisboa, Lisbon, Portugal

**Keywords:** COVID-19, sleep habits, quality of life, mental health, sleep disorders, older people

## Abstract

**Aim:**

This study aimed to describe sleep habits, quality of life and psychosocial aspects in older people and analyze associated differences considering the time before COVID-19 pandemic and during its first wave in Portugal.

**Methods:**

Online survey used for data collection received answers from 914 elderly (age range 65 – 90y), from April to August 2020.

**Results:**

Symptoms of self-perception of depression, anxiety, irritability and economic problems were not prominent in the elderly, except for worries concerning uncertainty about the future. There was no difference in sleep duration before and during the pandemic, although there was a worsening of some aspects related to sleep, such as sleep quality, sleep efficiency, awakening quality, sleep latency and nocturnal awakenings. Gender comparisons showed a higher vulnerability in women. Some morbidities got worse during the pandemic among the elderly, such as Insomnia, Headaches, Depression, Tinnitus, among others.

**Conclusion:**

Even though our data suggest that the pandemic did not have a great impact on quality of life, sleep quality and psychosocial aspects in the elderly, they were still affected by the worsening of their health conditions, including sleep and morbidities. Some behaviors may act as protective factors in this population, such as walking and keeping contact with others, as well as other aspects like financial stability, high level of education and family support, as they can help them to cope better with difficulties.

## Introduction

In late 2019, originated in Wuhan, China, a new coronavirus - severe acute respiratory syndrome coronavirus 2 (SARS-CoV-2), would be responsible for the worldwide pandemic of COVID-19 (Zhu et al., 2020). So far, this disease has killed 3,966,998 people around the world (Worldmeters, 2020, 22th June, 2020) and it is particularly dangerous for elderly people (Mueller et al., 2020). Apart from physical sufferings, this unpredictable infectious disease has also caused universal anxiety and distress, which are natural psychological responses to a randomly and lengthy changing condition.

The aged population is more prone to suffer severe forms from coronavirus infection (Brooke and Jackson, 2020; Cardinali et al., 2020). In those over 65, COVID-19 infection has a higher probability of progressing to pneumonia, lung consolidation, cytokine release syndrome, endothelins, coagulopathy, multiple organ failure, and death (Mueller et al., 2020). Some other diseases, such as hypertension, diabetes, obesity, cardiovascular disease, and respiratory system disease, are strongly associated with COVID-19 and contribute to the severity of the cases (Mueller et al., 2020; Neumann-Podczaska et al., 2020).

Health authorities and governments warned that older people are at a higher risk of severe symptoms and of fatal outcomes (Brooke and Jackson, 2020). Therefore, in many countries around the world massive lockdowns were imposed, with the associated home confinement affecting especially the elders. In addition to the physical damage COVID-19 can bring, during the pandemic the sustained and mandatory isolation could possibly negatively affect their psychological well-being and mental health, besides worsening feelings of loneliness (Briguglio et al., 2020). In modern societies social isolation and loneliness are a daily life feature of the elders (Fakoya et al., 2020).

Moreover, mental health conditions, such as depression, stress, and anxiety can lead to increased pro-inflammatory reactions, decreased resistance to infection (Perrotta et al., 2020), and cognitive decline (Landeiro et al., 2016). Feelings of fear can also be present due to their disease vulnerability (Li et al., 2020). In addition to those factors already mentioned, atypical homework schedules (Cardinali et al., 2020) or even the lack of a schedule are factors that may contribute to stress levels.

Stressful events in life have the potential to threaten one’s psychological and physical well-being (Morin et al., 2020), and more so among those with higher vulnerability, as in the elderly. The quarantine can produce mass hysteria, anxiety, and distress, due to many factors, like the sense of loss of control, uncertainty about the future, fear of contracting and spreading the infection to family members, separation from loved ones, inadequate and insufficient information, social-economic distress, among many others (Brooks et al., 2020; Dubey et al., 2020). Other aspects, such as false information, alarming news, and excess of time thinking about the outbreak, has been identified as stressors (Huang and Zhao, 2020).

Problems with sleep, like insomnia, sleep loss, and poor sleep quality have been a widespread complaint in this pandemic (Cellini et al., 2020). Considering that the prevalence of sleep disorders and sleep-related complaints in the elderly often ranges from 20 to 50% around the world (Gulia and Kumar, 2018), it is important to understand if, during the pandemic, these problems worsened.

Regulation of sleep occurs by two main systems: the homeostatic (related to the duration of wakefulness), and the circadian, which is controlled by daylight exposure, and other environmental timekeepers (Vadnie and McClung, 2017; Morin et al., 2020). During the confinement, the time spent outdoors, especially for the elders, was diminished, which may have caused deregulation of the circadian system (Morin et al., 2020). Besides, melatonin levels reduce with age, and higher exposure of individuals to light at night can also disrupt circadian rhythmicity (Cardinali et al., 2020).

The sleep pattern changes gradually as part of the normal aging process. Older people usually have difficulty in falling asleep, a shorter total sleep time, due to frequent arousals, a lower percentage of REM sleep, and a tendency to sleep and wake up earlier than younger adults (Gulia and Kumar, 2018).

Sleep quality is related to the quality of life (Zhi et al., 2016) and it plays an essential role in mental, psychological, and physical health (Franceschini et al., 2020; Morin et al., 2020). Moreover, as sleep is also involved with immune function (Irwin, 2019), bad sleep quality or even one night of bad sleep can lower immune defense and may be a risk factor for COVID-19 infection, in addition to hindering the recovery process.

Quality of life understood as a level of general well-being (Miniszewska et al., 2020) was also investigated in the elderly during the pandemic. However, the results are contradictory. Whereas one study found an association between older age and low levels of quality of life (Nguyen et al., 2020), another one found a good quality of life in this population (Bidzan-Bluma et al., 2020).

This study aimed to describe sleep habits, quality of life and psychosocial aspects in older age and evaluate if there were any differences in those aspects prior to and during COVID-19. It is important to understand at which level these aspects can contribute to the prevention and control of the disease. We hypothesized that during the pandemic, the elderly would have moderate levels of anxiety and concerns, as well as changes in health conditions and sleep habits compared to the period before the pandemic.

## Materials and Methods

### Setting and Study Population

The survey, as a whole, was specifically developed for this research project but it used as background knowledge the questionnaires routinely applied to the patients of CENC - Sleep Medicine Center and the literature concerning health and risk related habits, sleep schedules and associated habits and comorbidities.

The survey was made online by Survey Legend^®^ platform and it was targeted to also include elderly, from 65 years old. Surveys were anonymous, allowing data analysis and statistical use and it was released during the 1st COVID-19 wave in Portugal, from April to August 2020. The overall project was approved by CENC’s Ethical Committee 1/2020. The study was announced in the CENC’s website^1^, in the Facebook pages of the team participants, at the websites of the supporting professional associations (Medical, Nurses, Psychologists and Pharmaceutics) and by the sleep laboratories involved in the study, in the site of the supporting professional associations and *via* letters to most of the trade unions. There was no funding, public or private, no conflict of interests.

A total of 914 elderly from 65 to 90 years old completed the questionnaires. The study included data obtained in Portugal (Continent and Islands: Madeira and Azores). The inclusion criteria were survey answering. The exclusion criteria were not accepting statistical anonymous data use, too uncomplete surveys, obvious inconsistent answers (i.e., being on sick leave at 99 years of age).

### Questionnaires

On the first page of the survey, there was an explanation of the purpose, authors identification, approval by the Ethical Committee, names of a contact person in case of need, and supporting entities.

The study had 167 questions, subdivided into topics, as follows:

(a) Sociodemographic Characteristics - included questions about age, gender, civil status, and education level.
(b) Characteristics from the Lockdown and the Calamity (lockdown relief period) periods – questions requiring yes/no answers were asked for: voluntary confinement, home confinement and reasons to go out, including shopping, sports, walking, gardening, visiting family, visiting friends, taking children out, going to pharmacy and other reasons. For the Calamity period, the same yes/no questions were asked, with the addition of “hairdresser” and “restaurants” as reasons to go out.
(c) Characteristics of housing during confinement – included questions about type of house, house location and how many people were living in the house.
(d) COVID-19 infection in self and family – included questions about infection, infection severity, and death of someone by COVID or other cause.
(e) Psychosocial factors and Emotional Health – participants were asked to evaluate in a scale from 1 to 10 how they were living confinement, and how they perceived their levels of depression, anxiety, irritability, economic problems, and worries concerning uncertainty.
(f) Health Status and Morbidities - included yes/no questions about existing chronic diseases, such as Epilepsy, Hypertension, Parkinsonism, Fibromyalgia, Cardiovascular disease, Stroke, Respiratory diseases, allergies, Diabetes, Cancer, among others. The same procedure was used to ask the participants which comorbidities worsened, which morbidities improved, and which did not change during COVID.
(g) Sleep Habits – participants were asked about bedtime and time to get up (both on weekdays and on weekends), sleep duration (self-reported in hours), sleep latency (self-reported in minutes), awakenings (self-reported in number of awakenings during the night), sleep quality and sleep awakening quality (evaluated in a VAS 1-10, meaning the higher the score, the poorer the sleep/awakening quality) and sleep efficiency (self-reported sleep duration/sleep time *100, expressed as a percentage) pre and during COVID.
(h) TV viewing “Prior” and “During” COVID - yes/no questions, both for before and during COVID, namely for the type of TV programs watched during this time.

### Data Analysis

Qualitative variables were described by absolute (*n*) and relative frequencies (%), while quantitative variables were calculated by the mean and standard deviation. Normality of the data was tested by Kolmogorov-Smirnov test. Most continuous variables had a normal distribution. Comparisons were done using *t*-test, ANOVA and χ^2^-test, as appropriate for each type of data. Pearson correlation was used to analyze the correlation between variables and statistical significance was assessed with an alpha level of 0.05. The analysis was made in IBM SPSS Statistics 27.0.

## Results

Demographic data showed a male prevalence among the elderly who answered the survey, representing 59.3% against 40.7% of women. The average age of the elderly was 69.9 years, and the mode was 65 years. There were significantly more men than women participating in the study. Most of the elderly being married (71, 4%) and most of them had a graduate degree or higher (60, 6%) (Table 1).

**TABLE 1:** Demographic characteristics in the elderly.

		Elderly > 65 – 90y	
		Count	%
Gender	Male	542	59.3%
	Female	372	40.7%
Civil Status	Married	653	71.4%
	Bachelor	36	3.9%
	Widow	82	8.9%
	Divorced	113	12.3%
	Union	28	3.0%
Educational Level	Primary	59	6.5%
	Secondary	169	18.5%
	Bachelor degree	71	7.9%
	Graduate	434	48.0%
	Master	70	7.7%
	Ph.D.	44	4.9%

Regarding housing characteristics during the lockdown, most of the elderly stayed in their own home (89, 4%); most of the houses were located in the city (70%); and most of the elderly stayed at least with one more person as a companion during lockdown (only 15, 9% were alone) (Table 2).

**TABLE 2:** Housing characteristics of the elderly during Lockdown period.

		Elderly > 65	
		Count	%
Type of house	Flat	485	53.1%
	House	261	28.6%
	Apartment	74	8.1%
	Farm	37	4.1%
House location	City	570	62.4%
	Town	159	17.4%
	Village	81	8.9%
	Degraded zone	1	0.1%
Number of people in the house	Alone	145	15.9%
	One more	496	54.3%
	Two more	128	14%
	Three more	44	4.8%
	Four more	23	2.5%
	Five or more	33	3.6%

Most participants in this study were not infected by COVID-19 (χ^2^ = 11.698; *p* = 0.02), representing 97.9% of them. Of the 2.1% infected, 0.6% were asymptomatic, 1% had mild symptoms, 0.1% had pneumonia but stayed at home, and only 0.3% required hospitalization. Comparison of those infected with those not infected showed no differences in self-perception of depression (*Z* = 0.257; *p* = 0.612) anxiety (*Z* = 0.624; *p* = 0.430), irritability (*Z* = 1.083; *p* = 0.298), economic problems (*Z* = 0.667; *p* = 0.414), worries (*Z* = 1.197; *p* = 0.274), and worsening morbidities (*Z* = 0.637; *p* = 0.425).

Only 10.6% reported knowing a friend or familiar who got COVID-19, whilst 0.4% said one of these infected people died by the virus, and 8.1% reported a family member or friend who died by another cause during the same period.

### Psychosocial Factors

In the psychosocial factors section, participants were asked about self-perception of depression symptoms, anxiety, irritability, economic problems, and worries concerning uncertainty, through a 1 to 10 scale response (1 meaning no concern at all and 10 maximum concern). Elderly showed low levels of these symptoms, except concerning ‘uncertainty’, in which 50.4% answered from 6 to 10, meaning that half of the sample had moderate to strong feelings of uncertainty about the future. Table 3 shows the descriptive from this section.

**TABLE 3:** Psychosocial factors of the elderly.

	Min	Max	Mean (Sd)	Variance
How are you living confinement?	1	10	6.74 (1.9)	3.67
How is your depression?	1	10	3.42 (2.3)	5.42
How is your anxiety?	1	10	3.89 (2.4)	5.92
How is your irritability?	1	10	3.72 (2.3)	5.66
How are your economic problems?	1	10	2.85 (2.0)	4.38
How are your worries concerning uncertainty?	1	10	5.57 (2.5)	6.47
How is the frequency of your sexual activity?	1	10	3.13 (2.0)	4.33

*Min, Minimum response score; Max, Maximum response score; Sd, standard deviation.*

T-test comparison was made to analyze differences in psychosocial factors considering gender. Results showed significant differences regarding self-perception of depression and anxiety, as can be seen in Table 4. Women showed higher levels of subjective symptoms of depression and anxiety during the pandemic compared to men.

**TABLE 4:** Gender/Education level comparisons and psychosocial factors in the elderly.

	Mean				Mean								
	M	F	*t* (df)	*p*	Prim	Second	Prof	Bach	Grad	MSc	PHD	Z	*p*
Depression	3.05	3.97	−5.64 (837)	< 0.001	4.39	3.64	3.69	3.66	3.24	3.14	2.36	4.154	< 0.001
Anxiety	3.52	4.43	−5.32 (834)	< 0.001	4.8	4.21	3.64	4.16	3.76	3.63	2.69	3.969	0.001
Irritability	3.51	4.01	−3.01 (836)	0.190	4.43	3.98	3.65	4.17	3.46	4.02	2.81	3.581	0.002
Economic problems	2.78	2.96	−1.28 (836)	0.130	3.83	3.14	3.04	2.91	2.52	3.13	2.61	4.537	0.001
Worries	5.40	5.81	−2.28 (836)	0.800	6.35	5.34	5	6.06	5.62	5.61	4.71	2.705	0.013

*M, Male; F, Female; df, degrees of freedom; Prim, Primary; Secon, Secondary; Prof, Professional; Bach, Bachelor; Grad, Graduate; MSc, Master.*

Analyses were made considering educational level and psychosocial factors of subjective depression, anxiety and irritability, economic problems and worries. One-way ANOVA showed that there is an effect of educational level on these variables (*Z* = 4.154, *p* < 0001; *Z* = 3.969; *p* = 0.001; *Z* = 3.81; *p* = 0.002; 4.537; *p* = 0.001; 2.705; *p* = 0.013, respectively). Considering the variable depression, those with primary education differ from the group with graduation (*p* = 0.012) and Ph.D. (*p* < 0.001), just as the group with secondary education also differs from the group with Ph.D. (*p* = 0.028). Considering the anxiety variable, the group with a Ph.D. differs from the group with primary education (*p* < 0.001), secondary (*p* = 0.006) and bachelor (*p* = 0.039). Finally, considering the irritability variable, only the primary education group differs from the group with a Ph.D. (*p* = 0.018). In all these comparisons, the Ph.D. group had lower levels of perceptive depressive symptoms, anxiety symptoms, and irritability compared to the other groups. Economic problems differed between graduate and both secondary and primary education; and worries between Ph.D. and primary education (see Table 4).

Some of these variables were also correlated with sleep parameters, such as sleep quality, sleep latency and awakening quality. As can be seen in Table 5, almost all correlations were negative and statistically significant, meaning that the lower the levels of subjective depression, anxiety and irritability, the better the levels of sleep and awakening quality. Correlations with sleep latency showed higher levels of subjective depression and anxiety related to higher sleep latency. However, all these correlations although significant were weak.

**TABLE 5:** Correlations between psychosocial factors and sleep parameters in the elderly.

	Sleep quality		Awakening quality		Sleep latency	
	*R*	P	*R*	P	*R*	P
Depression	−0.391	<0.0001	−0.410	<0.0001	0.297	< 0.0001
Anxiety	−0.415	<0.0001	−0.419	<0.0001	0.255	< 0.0001
Irritability	−0.375	<0.0001	−0.400	<0.0001	-	-

### Sleep Habits

Before the pandemic, the elderly used to sleep from 6-7 hours during the weekdays, and 7-8 hours during the weekends. During the pandemic, they continued with the same sleep schedule and there were no changes regarding sleep duration. Table 6 shows some sleep parameters and the differences between the pre- and mid-pandemic periods and this data is also presented. It can be noted that there was a negative change regarding some parameters, such as sleep latency, sleep quality, sleep awakening quality, awakenings, and sleep efficiency. These results reveal that the pandemic period had an impact on elders’ sleep.

**TABLE 6:** Comparisons of sleep parameters in the elderly before and during COVID.

	Mean			
	Before COVID	During COVID	*t* (df)	*p*-value
Sleep Duration	6.94	7.03	−1.43 (677)	0.153
Time into bed	0.13	0.18	−1.162 (719)	0.246
Time out of bed	8.0	8.2	−4.41 (748)	< 0.0001
Sleep latency	24.5	30.9	−7.34 (639)	< 0.0001
Awakenings	2.2	2.5	−3.23 (551)	< 0.0001
Sleep Quality	6.5	6.1	8.84 (693)	< 0.0001
Sleep Awakening Quality	6.8	6.4	7.46 (685)	< 0.0001
Sleep Efficiency	85.1	83.1	4.35 (602)	< 0.0001

df, degrees of freedom. Sleep duration: reported in hours; sleep latency: self-reported sleep latency in minutes; awakenings: self-reported number of awakenings during the night; sleep quality and sleep awakening quality: self-reported quality, with higher scores indicating better quality; sleep efficiency: self-reported sleep duration/sleep time *100, expressed as a percentage.

Gender differences were also measured considering the same sleep parameters, as can be seen in Table 7. Women exhibited higher sleep latency and more awakenings during sleep, as well as lower sleep efficiency, sleep quality and sleep awakening quality, compared to men.

**TABLE 7:** Gender comparisons and sleep parameters in the elderly.

	Mean			
	Male	Female	*t*(df)	*p*-value
Sleep duration	7,0	7,0	0,35(685)	0.200
Sleep latency	23,9	41,8	−6,73(643)	< 0.0001
Awakenings	2,2	3,1	−2,97(586)	< 0.0001
Sleep efficiency	84,5	80,7	2,94(622)	< 0.0001
Sleep quality	6,6	5,4	7,58(693)	< 0.0001
Sleep waking quality	6,8	5,9	5,90(688)	< 0.0001

df, degrees of freedom. Sleep duration: reported in hours; sleep latency: self-reported sleep latency in minutes; awakenings: self-reported number of awakenings during the night; sleep quality and sleep awakening quality: self-reported quality, with higher scores indicating better quality; sleep efficiency: self-reported sleep duration/sleep time *100, expressed as a percentage.

### Health Status

In Health Status and Morbidities section, we found a variety of diseases that the elderly had since before the pandemic. The morbidity index (MI) is the sum of all referred morbidities at baseline with respect to COVID-19 in terms of worsening (morbidities worsening index (MWI)). In Table 8 the MWI, shows the effect of pre-pandemic morbidities upon global health status evaluated by the Morbidity Worsening Index. It is clear that most disorders did not have significant effects upon morbidities worsening, while in some of them the effect was clear, namely, Insomnia, Headaches, Depression, Anxiety, Burn out, Dizziness and Tinnitus.

**TABLE 8:** Morbidities Pre COVID and Morbidities worsening in the elderly.

Morbidities PreCOVID		Disorder not present	Disorder present	Morbidity Worsening Index (MWI)	
	% (*n*)	MWI mean	MWI mean	Z	*p*-value
Insomnia	14.4 (98)	1.36	2.19	17.052	< 0.001
Sleep Apnea	41.1 (280)	1.45	1.51	0.171	0.679
Headaches	3.1 (28)	1.44	2.77	10.910	0.001
Diabetes	3.1 (28)	1.46	2.0	2.010	0.157
Hypertension	15.5 (138)	1.44	1.68	1.614	0.204
Cardiovascular	5.1 (45)	1.46	1.76	0.926	0.336
Respiratory diseases	4.9 (45)	1.48	1.52	0.013	0.908
Allergies	5.3 (47)	1.46	1.78	1.227	0.268
Gastrointestinal	3.1 (21)	1.46	2.05	1.998	0.158
Endocrinology metabolic	1.9 (17)	1.48	1.50	0.002	0.966
Rheumatologic diseases	2.1 (19)	1.46	2.00	1.430	0.232
Orthopedic diseases	2.2 (20)	1.50	0.83	2.195	0.239
Autoimmune disease	1.6 (14)	1.46	2.21	2.204	0.138
Cancer	4.2 (37)	1.47	1.65	0.288	0.591
Urologic disease	2.1 (19)	1.48	1.50	0.002	0.963
ENT problems	1.1 (10)	1.47	1.78	0.232	0.630
Ophtalmologic diseases	2.4 (22)	1.46	2.05	1.834	0.176
Chronic Pain	2.2 (20)	1.47	1.90	1.041	0.308
Fatigue	3.3 (29)	1.46	2.00	2.162	0.133
Dizziness	1.7 (15)	1.63	4.07	37.507	< 0.0001
Tinnitus	2.6 (18)	1.61	4.72	76.328	< 0.0001
Burnout stress	4.8 (43)	1.41	2.93	19.405	< 0.0001
Anxiety	7.5 (67)	1.37	2.71	26.783	< 0.0001
Depression	8.8 (80)	1.38	2.47	18.750	< 0.0001

In Table 9, it is possible to see lockdown housing and behaviors comparisons with the Morbidity Worsening Index. Behaviors such as shopping, walking and gardening during lockdown acted as protective factors for not worsening morbidities during the pandemic period.

**TABLE 9:** Lockdown characteristics and the Morbidity Worsening Index.

		Morbidity Worsening Index			
				
		No	Yes	Z	*p*
Lockdown in house	Count	506	181	1.677	0.196
	Mean	1.43	1.64		
Lockdown shopping	Count	277	410	4.034	0.045*
	Mean	1.66	1.37		
Lockdown children out	Count	679	8	0.344	0.558
	Mean	1.48	1.88		
Lockdown Sports	Count	671	16	2.689	0.102
	Mean	1.47	2.25		
Lockdown Walking	Count	452	235	3.996	0.046*
	Mean	1.59	1.29		
Lockdown visit family	Count	666	21	0.045	0.831
	Mean	1.48	1.57		
Lockdown friends	Count	680	7	0.464	0.496
	Mean	1.49	1.00		
Lockdown gardening	Count	593	94	3.898	0.049*
	Mean	1.54	1.13		
Lockdown pharmacy	Count	420	267	0.264	0.608
	Mean	1.51	1.44		
Lockdown working	Count	562	1.45	0.829	0.363
	Mean	1.45	1.62		

Morbidities Worsening Index are for the post-COVID situation; Z-scores indicate the differences between the groups. *Statistically significant difference, p < 0.05.

### Lockdown Characteristics

Regarding lockdown characteristics, they were asked about reasons to go out during the lockdown period.

Comparisons were made between some lockdown characteristics and psychosocial factors. The independent *t*-test showed that, on average, the elderly who went out for a walk during the lockdown had lower levels of self-perception of depression (*t* = 2,73; *p* = 0.007), anxiety (*t* = 2,09; *p* = 0.037) and irritability (*t* = 2,08; *p* = 0.038). The same comparisons were made for the variables that had a higher percentage of “yes” answers, such as shopping, gardening, going to pharmacy and working, but no significant differences were found, as can be seen in Table 10.

**TABLE 10:** Lockdown behaviors and comparisons with psychosocial factors among the elderly.

			Depression		Anxiety		Irritability	
			*T*	*p*-value	*t*	*p*-value	*T*	*p*-value
Lockdown Shopping	No	39.6%	0.68 (837)	0.496	0.80(834)	424	0.13(838)	0.894
	Yes	60.4%						
Lockdown Walking	No	66.9%	2.73 (837)	0.007*	2.09(834)	0.037*	2.08(838)	0.038*
	Yes	33.1%						
Lockdown Gardening	No	87.1%	2.43(837)	0.061	1.56(834)	0.118	1.03(838)	0.301
	Yes	12.9%						
Lockdown Pharmacy	No	60.3%	−0.89(837)	0.374	−0.40(834)	0.686	−0.72(838)	0.471
	Yes	39.7%						
Lockdown Working	No	82.8%	1.74(837)	0.082	0.551(834)	0.581	0.412(438)	0.680
	Yes	17.2%						

*Statistically significant difference, p < 0.05.

The use of television for entertainment and information continued frequent during the pandemic. The program influences upon psychological variables were as follows: Those watching TV news had lower anxiety values (Z = 3.878, *p* = 0.049) and lower economic problems (Z = 4.574, *p* = 0.033); those watching soap operas had lower anxiety levels (Z = 5.448, *p* = 0.020) and those watching TV reports had lower self-reported depression levels (Z = 5.080, *p* = 0.016); TV series/movies, TV contexts and TV documentaries during the pandemic had no significant effects upon psychological and economic variables. In spite of these beneficial effects, not watching TV was associated with lower self-reported depression levels (*Z* = 4.656, *p* = 0.031).

## Discussion

This study aimed to characterize psychosocial aspects, health-related problems and sleep habits in the elderly and to compare them with the period before the COVID-19 pandemic to analyze possible changes caused by the pandemic.

Overall, our study found a high level of education within the respondent elderly. Almost half of them reported university education (48%). A study made by Bidzan-Bluma et al. (2020) also found a high educational level between elderly who took part in their study, and they believe that this might be associated with quality of life, well-being, and life satisfaction. We found in our sample that the ones with a Ph.D. showed significantly lower levels of perceived symptoms of depression, anxiety and irritability compared to the ones who had only primary education. Thus, the high educational level acted as a protective factor for mental health in this elderly population. These results are in line with other study that presented lower education associated with low levels of quality of life in the older age (Huong et al., 2017).

It is also interesting to notice that there were more men than women in this study, which is not so common considering research with use of questionnaires. Perhaps it is a point to explain the high level of formal education in the sample. Although nowadays women match or overcome men’s educational achievements (Bobbitt-Zeher, 2007), for several decades, there were gender differences considering education and career development (McWhirter, 1997). For instance, females were more likely to perceive discrimination and child rearing as greater barriers to career development than did males (Swanson and Tokar, 1991).

Symptoms of psychological distress have already been investigated in the quarantined population, generally reporting a high prevalence of psychological symptoms, emotional disturbance, depression, and post-traumatic stress symptoms (Yoon et al., 2016). As the elderly are exposed to a greater risk of developing more severe symptoms of COVID-19, we expected that participants in our study would have high levels of anxiety or depression.

Nevertheless, they showed low levels of self-perceived symptoms of anxiety, depression, and irritability. Although these results were unexpected, they agree with other studies made during this current pandemic. Huang and Zhao (2020) found high levels of anxiety generalized disorder and poor sleep quality in the Chinese public during the outbreak, but the younger (aged < 35 years) were more likely to develop anxiety symptoms compared to the older ones. A Brazilian study made by Barros et al. (2020) also found more symptoms of sadness, depression, and anxiety in young adults compared to older adults. Levels of fear, anxiety and depression were equally investigated by Pinto et al. (2020) and they were less pronounced in the elders than in the younger.

These results are newsworthy, since old adults are at a greater risk of developing the severe COVID-19 outcomes. Some authors believe that as the elderly are less affected by pandemic financial problems since they were not facing the threat of a job loss, this can be a reason for not feeling so anxious or depressed (Barros et al., 2020; Bidzan-Bluma et al., 2020). In our study, participants were also asked about financial problems and the great majority of them showed no concern about this issue. Additionally, even during the lockdown period, the elderly did not stay completely at home, but many of them went out shopping and going for a walk, activities that were allowed provided the social detachment and the use of a mask was maintained. These behaviors, although simple, allowed for the possibility of not being at home all the time.

Our results showed that walking served as a way to reduce the self-perceptive symptoms of depression, anxiety and irritability, which may have contributed to reduce feeling of isolation. Besides, there was the frequent use of television for entertainment, which may have helped them alleviate anxiety and stress symptoms. Activities focused on leisure and/or skill development are usually recommended to reduce loneliness and improve well-being in the elderly (Pettigrew and Roberts, 2008; Girdhar et al., 2020).

Although we found low levels of subjective symptoms of anxiety and depression in our sample, women showed higher vulnerability compared to men. Some studies have already shown that women are more likely to experience psychological distress and worsening of health status than men due to several factors, such as childcare responsibilities (Zamarro et al., 2020) and greater domestic work load (Alon et al., 2020). Women have a higher prevalence of other risk factors, such as anxiety and depressive disorders, that were intensified during the pandemic period (Bucciarelli et al., 2022).

Elders used to be encouraged to interact with family, friends, neighbors, and community in their daily living (Chen, 2020). However, during the COVID-19 pandemic, they were stimulated to do the opposite to prevent infection. Elders’ isolation has been a concern among specialists since the beginning of the pandemic, as social isolation among older adults could lead to negative mental and physical health consequences (Armitage and Nellums, 2020). Manageable behaviors, such as walking, was associated with low levels of perceived depression, anxiety and irritability, so it is believed that this behavior contributed to the well-being of the elderly. In tandem with walking, going out for shopping and gardening were behaviors that acted as protective factors for the worsening of morbidities. These results are interesting, as using free time for relaxation activities and staying busy has been shown to be protective wellness behaviors during this pandemic (Ingram et al., 2020; Paiva et al., 2021), specially for older people (Jiménez-Pavón et al., 2020).

Another considerable reason that may have contributed to the low levels of self-reported depression symptoms and anxiety is high elderly resilience when facing difficulties in their lifetime (Fontes and Neri, 2015; Serafini et al., 2020; Grossman et al., 2021). Resilience in the elders is considered a predictor for mental health and better health outcomes (MacLeod et al., 2016).

Resilience along with social support are aspects that may have helped them to cope with the situations caused by the pandemic. Elderly social support has been associated with health-related quality during the pandemic (Levkovich et al., 2021). In our study, the great majority of the elderly stayed at home with at least one family member. Being more at home, despite all the associated problems previously discussed, can also contribute to feelings of self-responsibility, and the citizenship reward in preventing disease spread.

Furthermore, they did not stay completely isolated, as they kept in contact with their family. This may have helped to decrease the feeling of isolation, which was reflected in the low levels of subjective depression, anxiety, and sleep problems and enhances the importance of family bonds. In a study with Finnish elderly with a risk of developing cognitive impairment, the authors did not find considerable changes in lifestyle and behaviors, but they pointed that those living alone were more susceptible to negative changes (Lehtisalo et al., 2021).

TV viewing of news, reports and soap novels had some beneficial effect upon anxiety and depression; it is well recognized that TV is an important companion of the elders. However, it must be stressed that no TV was also associated with significantly lower depression levels. In adolescents and in adults, high levels of TV viewing (Paiva et al., 2021) are considered health risk behaviors.

Another important observation is the role of literacy in the general quality of life. In adults, usually, a high level of education is associated with good quality of life. However, a study made during this pandemic found higher literacy associated with poor quality of life (Nguyen et al., 2020). The authors believe that this unexpected result may be explained by the fact that this group was the one who continued going to work and/or dealing with the family burden (taking care of children, shopping for elderly parents during the pandemic, among others). In the elderly, though, higher education can act as a protective factor (Bidzan-Bluma et al., 2020; Nguyen et al., 2020). In our study, a sizable part of the elderly had high educational level and as most of them were retired, these factors may also have contributed to perceived good quality of life, low levels of depression, anxiety, irritability, good sleep quality, resilience, and a better understanding of coping strategies against COVID-19 (Chen, 2020).

Sleep quality has also been investigated during this pandemic, and it is assumed that older age impacts negatively in sleep quality, as sleep changes along the aging process. Similar to aspects of depression and anxiety, in all those studies, there was a higher prevalence of sleep problems in younger adults in comparison to older adults (Barros et al., 2020; Bidzan-Bluma et al., 2020; Beck et al., 2021). A recent study characterized the impact of the COVID-19 pandemic on sleep and awakening qualities of a diversified population, including the elderly (Paiva et al., 2021). Elders reported a significantly better awakening quality compared to the younger, but no age differences in sleep quality were found. Both these aspects were associated with several factors and being older was one of the protective factors for poor awakening quality.

Grossman et al. (2021) found higher levels of COVID-19 related loneliness associated with higher levels of sleep problems among older adults. In another study, older age was considered a protective factor for difficulties in falling asleep, waking up too early in the morning, and non-restorative sleep reports (Pinto et al., 2020). Even though small, in our sample, we found a worsening of some sleep aspects, such as sleep quality, sleep awakening quality, sleep latency and sleep efficiency, between the pre- and mid- pandemic periods. Besides, women reported worse rates compared to men in all these aspects, except sleep duration. These results are in line with the study from Franceschini et al. (2020), which found female gender as a risk factor during the outbreak restrictions in Italy.

Low sleep quality has been found in individuals during the lockdown, and it is directly associated with high levels of anxiety, stress (Xiao et al., 2020), and depression symptomatology (Franceschini et al., 2020) in the general population. Conditions such as psychological distress, stress, anxiety, depressive symptoms, death of someone close due to COVID-19, low levels of social capital, and fear of getting infected/spreading the infection are frequently associated with poor sleep quality during COVID-19 pandemic (Bigalke et al., 2020; Franceschini et al., 2020; Gupta et al., 2020; Xiao et al., 2020).

As discussed above, our sample did not show high levels of anxiety, depression, irritability, worries or economic problems, and these aspects were weakly correlated to sleep quality, sleep latency and awakening quality. This subtle association may be related to the low variety of psychosocial aspects, which may have contributed to the maintenance of good sleep quality.

In the present study, we also found a worsening of morbidities during the pandemic. This worsening was clearly higher in subjects who previously suffer from insomnia, headaches, depression, anxiety and burnout, dizziness and tinnitus. These differences stress the impact of mental health, especially in stressful situations, the relevance of insomnia and chronic headaches and, most of all, the importance of minor complaints such as dizziness and tinnitus, which are often discarded by the medical community. Although we did not find an association between worsening of morbidities in other organic medical disorders, such as cardiovascular disease, depression is a risk factor for cardiovascular disorders, which is one of the most dangerous morbidities for the worsening of COVID-19 (Mattioli et al., 2020; Samlani et al., 2021).

We have no data to stand whether these elderlies were left unattended during the lockdown period, but some authors state that this fragile group was neglected by the health system (Lloyd-Sherlock et al., 2020). Whether this happened or not, it is extremely important to take care of people who have not been infected by the virus but have other diseases that need constant monitoring. Special care should be taken in patients with insomnia, headaches, depression, anxiety and burnout, dizziness and tinnitus.

There were no differences in worsening morbidities and psychological variables in the small group of COVID infected patients when compared with non-infected subjects. This negative finding is certainly due to the low number of subjects infected and severity variability.

This study had certain limitations. Firstly, it is based on participants’ subjective self-report questionnaires, so it is reasonable to expect a sampling bias. Secondly, the data refers only to the period in which they were collected, and figures may change over time, given the dynamics of the virus, and the extended period of quarantine in Portugal. Also, as this study was made online, the survey only reached the elderly who had access to the internet, which may not be representative of the elderly population.

## Conclusion

Our findings suggest that the pandemic had a low negative impact on quality of life, sleep quality and psychosocial aspects in the elderly from Portugal. However, they suffered worsening of some health conditions and sleep parameters modifications. High level of education acted as protective factor in this population. Furthermore, most of them were not completely isolated at home during the period when the survey occurred, what we believe was something that helped them to lessen the sense of social isolation. Besides, behaviors of self-care like walking helped them to maintain their quality of life. Therefore, this population group had many resources that help them to cope with psychosocial vulnerabilities brought by this pandemic. However, their frailty is genuine, especially in female gender, and it is important to pay attention to the vulnerable groups and provide them with strategies to reduce loneliness and increase resilience and social support, as they act as protective factors.

## Data Availability Statement

The original contributions presented in the study are included in the article/supplementary material, further inquiries can be directed to the corresponding author.

## Ethics Statement

The studies involving human participants were reviewed and approved by CENC’s Ethical Committee. The patients/participants provided their written informed consent to participate in this study.

## Author Contributions

KA and TP: study design. KA, TP, and EC: writing the draft, integration of the authors comments, and final manuscript. All authors contributed to the article and approved the submitted version.

## Conflict of Interest

The authors declare that the research was conducted in the absence of any commercial or financial relationships that could be construed as a potential conflict of interest.

## Publisher’s Note

All claims expressed in this article are solely those of the authors and do not necessarily represent those of their affiliated organizations, or those of the publisher, the editors and the reviewers. Any product that may be evaluated in this article, or claim that may be made by its manufacturer, is not guaranteed or endorsed by the publisher.

## Footnotes

1
www.isleep.pt
